# Clinical significance of HER2-low expression in early breast cancer: a nationwide study from the Korean Breast Cancer Society

**DOI:** 10.1186/s13058-022-01519-x

**Published:** 2022-03-21

**Authors:** Hye Sung Won, Juneyoung Ahn, Yongseon Kim, Jin Sung Kim, Jeong-Yoon Song, Hong-Kyu Kim, Jeeyeon Lee, Heung Kyu Park, Yong-Seok Kim

**Affiliations:** 1 Department of Internal Medicine, College of Medicine, The Catholic University of Korea, Seoul, Republic of Korea; 2 Department of Surgery, College of Medicine, The Catholic University of Korea, Seoul, Republic of Korea; 3 Department of Surgery, Ulsan University Hospital, University of Ulsan College of Medicine, Ulsan, Republic of Korea; 4 Department of Surgery, Kyung Hee University Hospital at Gangdong, Seoul, Republic of Korea; 5 Department of Surgery, Seoul National University College of Medicine, Seoul, Republic of Korea; 6 Department of Surgery, Kyungpook National University School of Medicine, Daegu, Republic of Korea; 7 Department of Surgery, Gachon University Gil Medical Center, Incheon, Republic of Korea

**Keywords:** Breast cancer, HER2-low, Prognosis

## Abstract

**Background:**

There is an increasing interest in HER2-low breast cancer with promising data from clinical trials using novel anti-HER2 antibody–drug conjugates. We explored the differences in clinicopathological characteristics and survival outcomes between HER2-low and HER2-IHC 0 breast cancer.

**Methods:**

Using nationwide data from the Korean Breast Cancer Registry between 2006 and 2011, 30,491 patients with stages I to III breast cancer were included in the analysis: 9,506 (31.2%) in the HER2-low group and 20,985 (68.8%) in the HER2-IHC 0 group. Kaplan–Meier and Cox proportional hazards regression survival analysis were used to compare breast cancer-specific survival between the two groups.

**Results:**

HER2-low breast cancer was more frequent in patients with hormone receptor-positive breast cancer than in those with triple-negative breast cancer. In patients with hormone receptor-positive breast cancer, HER2-low breast cancer was associated with fewer T4 tumors, higher histological grade, and a negative lymphatic invasion. In patients with triple-negative breast cancer, HER2-low breast cancer was associated with a high lymph node ratio and positive lymphatic invasion. HER2-low breast cancer was significantly associated with a lower Ki-67 labeling index. No significant difference was observed in overall survival between the two groups. HER2-low breast cancer showed significantly better breast cancer-specific survival than HER2-IHC 0 breast cancer, regardless of the hormone receptor status. In multivariate analysis, the impact of low HER2 expression on breast cancer-specific survival was significant only in triple-negative breast cancer (HRs, 0.68; 95% CI, 0.49–0.93; *P* = 0.019).

**Conclusions:**

These findings suggest that the biology and clinical impact of low HER2 expression can differ according to the hormone receptor status and support the need for further investigation on the understanding of the biology of HER2-low breast cancer.

**Supplementary Information:**

The online version contains supplementary material available at 10.1186/s13058-022-01519-x.

## Background

The discovery of human epidermal growth factor receptor 2 (HER2) marked a major milestone in the treatment of patients with breast cancer, and a phenomenal success story is still in progress [1–3]. Based on the results of early clinical trials using the first anti-HER2 targeted agent, trastuzumab, HER2-positive breast cancer has been defined as tumors with an immunohistochemistry (IHC) score of 3 + for HER2 staining or IHC score of 2 + with HER2 gene amplification by in situ hybridization (ISH) assay [4–6]. The remaining tumors have been classified as HER2-negative breast cancer. Recently, this dichotomous classification of HER2 status has been challenged to reclassify HER2-negative breast cancer into two categories: HER2-low (IHC score of 1 + or 2 + without HER2 gene amplification) and HER2-negative (IHC score of 0, no staining [HER2-IHC 0]) breast cancer [7]. This emerging concept started to gain attention with the results of recent clinical trials using novel anti-HER2 targeted agents, such as trastuzumab deruxtecan and trastuzumab duocarmazine [8–10]. Interestingly, these drugs showed antitumor efficacy in patients with HER2-low breast cancer as well as in those with HER2-positive breast cancer. This effect is explained by a high cytotoxic payload and potent bystander killing effect, which leads to the death of adjacent antigen-negative tumor cells, following the release of the cytotoxic payload from dead, antigen-positive cells [11]. On the other hand, this observation suggests that HER2-low and HER2-IHC 0 breast cancers may be different disease entities. However, to date, data on the clinical difference between the two groups are insufficient and a complete understanding of the biology of HER2-low breast cancer is lacking.

HER2-low breast cancer accounts for approximately 45% to 55% of all breast cancers, and heterogeneity exists according to the expression of hormone receptor (HR) [7]. Because the proportion of patients with HER2-low breast cancer is substantial, efforts to develop precision medicine strategies and improve survival of these patients can be meaningful. This requires understanding of the clinical characteristics and prognosis of these patients. In this study, we aimed to investigate the differences in clinicopathological characteristics and survival outcomes between patients with HER2-low breast cancer and those with HER2-IHC 0 breast cancer using nationwide data from the Korean Breast Cancer Registry (KBCR).

## Materials and methods

### Data collection and study population

The KBCR database is an online registry maintained by the Korean Breast Cancer Society involving more than 100 institutions in Korea. Before entering personal information into this registry, written informed consent was obtained from all patients. The following clinicopathological data were collected from the KBCR database: basic demographic characteristics (sex, age, body mass index [BMI], and menopausal status), family history of breast cancer, date of diagnosis of breast cancer, date of surgery, types of surgery, pathological TNM stage according to the American Joint Committee on Cancer staging system, pathological characteristics (histological types, tumor location, tumor size, number of positive lymph nodes [LNs], total number of LNs removed, histological grade, nuclear grade, lymphatic invasion, vascular invasion, estrogen receptor [ER] status, progesterone receptor [PR] status, HER2 status, and Ki-67 labeling index), neoadjuvant chemotherapy, adjuvant treatment (endocrine therapy, radiation therapy, and chemotherapy), survival, date of death, and cause of death. The lymph node ratio (LNR) was calculated as the number of positive LNs divided by the total number of LNs removed. This database does not contain information about tumor recurrence. Updated data on the date of death and cause of death were obtained from the Korean Central Cancer Registry, Ministry of Health and Welfare, Korea. The assessment of HER2 status was based on the HER2 testing guidelines of the American Society of Clinical Oncology/College of American Pathologists.

**Fig. 1:**
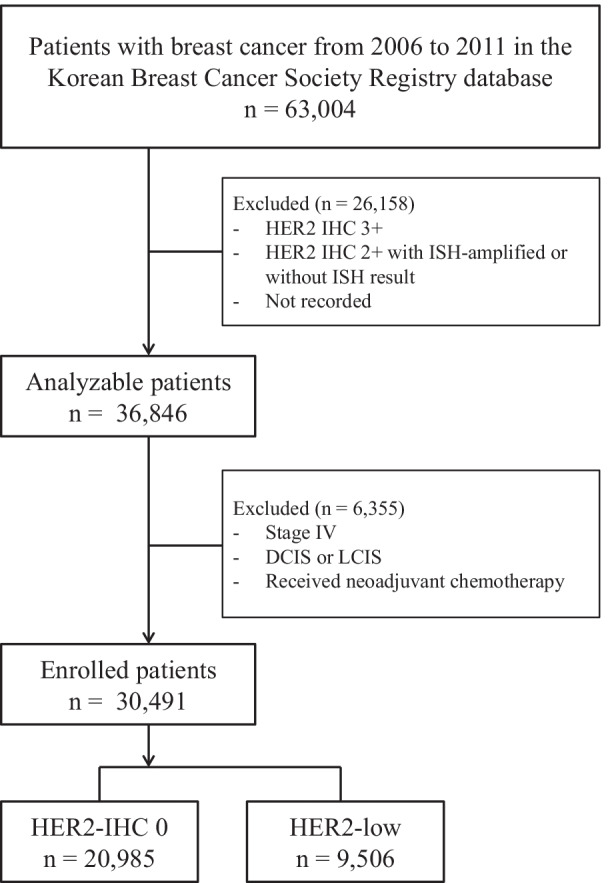
Flow diagram for the selection of patients in this study

The patient selection process for this study is summarized in Fig. 1. Among 63,004 patients diagnosed with breast cancer between January 1, 2006 and December 31, 2011, those with HER2-positive breast cancer, incomplete data on HER2 status, ductal carcinoma or lobular carcinoma in situ, stage IV cancer, and receipt of neoadjuvant chemotherapy were excluded. Finally, 30,491 patients were included in the analysis, including 20,985 (68.8%) patients with HER2-IHC 0 breast cancer and 9,506 (31.2%) patients with HER2-low breast cancer.

### Statistical analysis

Categorical variables were compared using the chi-square and Fisher’s exact tests. The significance of differences in continuous variables was evaluated using Student’s *t*-test. Survival curves were estimated using the Kaplan–Meier method, and the log-rank test was used to compare differences. Overall survival (OS) was defined as the time from the date of surgery to the date of death from any cause or the date of last follow-up. Breast cancer-specific survival (BCSS) was defined as the time from the date of surgery to the date of death caused by breast cancer or the date of last follow-up. Univariate and multivariate analyses were performed using the Cox proportional hazards regression models, and adjusted hazard ratios (HRs) with 95% confidence intervals (CI) were calculated. Additionally, to reduce the effects of imbalanced confounding factors between the groups, the inverse probability of treatment weighting (IPTW) using the propensity score was used. To estimate the propensity score, logistic regression model was applied, and propensity scores were calculated based on pathological stage, tumor size, LNs metastasis, LNR, histological grade, lymphatic invasion, vascular invasion, and Ki-67 labeling index. IPTW-adjusted Kaplan–Meier curves were also estimated. All data were analyzed using SPSS software (v. 18; SPSS, Chicago, IL, USA) and *P* < 0.05 was considered significant.

## Results

### Clinicopathological characteristics of the patients according to HER2 status

Of the total, 23,539 (77.3%) patients had HR-positive breast cancer and 6,934 (22.7%) had triple-negative breast cancer (TNBC). The proportion of HER2-low breast cancer was higher in patients with HR-positive breast cancer than in those with TNBC: 7,910 (33.6%) in HR-positive breast cancer and 1,594 (23.0%) in TNBC (*P* < 0.001). According to the HR status, the differences in clinicopathological characteristics between patients with HER2-low breast cancer and those with HER2-IHC 0 breast cancer are summarized in Table 1. The median age at diagnosis was higher for the HER2-low tumors compared to HER2-IHC 0 tumors within TNBC (*P* = 0.001), but no difference was observed between the groups in the proportion of young patients under the age of 40. Among patients with HR-positive breast cancer, premenopausal patients were more frequently observed in the HER2-low group (*P* = 0.001). The prevalence of overweight with a BMI ≥ 25 kg/m^2^ was higher in the HER2-low group for both HR-positive breast cancer and TNBC (*P* = 0.001 and 0.015, respectively). No difference was noted in the pathological stage (stages 1 to 3) between the two groups, but a tendency of more T1 tumors and fewer T4 tumors was observed in the HER2-low group (*P* = 0.041 for HR-positive, and 0.05 for TNBC, respectively). Among patients with TNBC, LNR was higher in the HER2-low group than the HER2-IHC 0 group (*P* = 0.018). Among patients with HR-positive breast cancer, tumors with histological grade 1 were more frequently observed in the HER2-IHC 0 group (*P* = 0.001). Among patients with TNBC, the prevalence of lymphatic invasion was higher in the HER2-low group than HER2-IHC 0 group (*P* = 0.001). However, the opposite result was observed for HR-positive breast cancer (*P* = 0.001). The frequency of tumors with a Ki-67 labeling index above 20% was higher in TNBC than HR-positive breast cancer (73.5% vs. 31.3%, *P* < 0.001). Regarding Ki-67 labeling index according to HER2 status, patients with low Ki-67 labeling index were more frequent in the HER2-low group than the HER2-IHC 0 group for both HR-positive breast cancer and TNBC, regardless of the Ki-67 cutoff value (*P* = 0.001 and 0.001, respectively).

**Table 1:** Clinicopathological characteristics of patients with HER2-low breast cancer according to hormone receptor status

Variables	All patients (N = 30,491)		*P* value	HR-positive (N = 23,539)^a^		*P* value	HR-negative (N = 6,934)		*P* value
	HER2_0	HER2_L		HER2_0	HER2_L		HER2_0	HER2_L	
Age, median (range), y^b^	48 (20–94)	49 (21–92)	.127	48 (20–94)	49 (23–90)	.267	48 (20–91)	50 (21–91)	.001
Age, No, (%), y^b^			.267			.462			.410
< 40	2,997 (14.2)	1,311 (13.8)		1,934 (12.4)	1,016 (12.9)		1,062 (19.9)	295 (18.5)	
≥ 40	17,985 (85.8)	81,76 (86.2)		13,682 (87.6)	68,77 (87.1)		4,268 (80.1)	1,297 (81.5)	
Sex, No. (%)			.021			.084			.547
Female	20,909 (99.6)	9,454 (99.4)		15,557 (99.5)	7,860 (99.4)		5,336 (99.9)	1,592 (99.9)	
Male	76 (0.4)	52 (0.6)		72 (0.5)	50 (0.6)		4 (0.1)	2 (0.1)	
Menopausal status, No. (%)^b^			.001			.001			.505
Premenopause	7,474 (68.6)	4,373 (72.5)		5,541 (70.6)	3,628 (74.3)		1,928 (63.3)	739 (64.4)	
Postmenopause	3,423 (31.4)	1,660 (27.5)		2,305 (29.4)	1,252 (25.7)		1,117 (36.7)	408 (35.6)	
BMI, No. (%), kg/m^2^			.001			.001			.015
< 25	16,176 (77.1)	7,087 (74.6)		12,104 (77.4)	5,921 (74.8)		4,059 (76.0)	1,164 (73.0)	
≥ 25	4,809 (22.9)	2,419 (25.4)		3,525 (22.6)	1,989 (25.2)		1,281 (24.0)	430 (27.0)	
Presence of FHx, No. (%)^b^	1,394 (8.5)	739 (9.5)	.010	1,003 (8.2)	612 (9.4)	.005	391 (9.4)	127 (10.0)	.500
Tumor location, No. (%)^b^			.447			.220			.546
Right	9,752 (50.0)	4,066 (49.9)		7,393 (50.7)	3,403 (50.0)		2,354 (48.1)	670 (49.8)	
Left	9,703 (49.8)	4,078 (50.0)		7,165 (49.1)	3,395 (49.9)		2,533 (51.7)	674 (50.1)	
Both	43 (0.2)	9 (0.1)		26 (0.3)	7 (0.1)		8 (0.2)	2 (0.1)	
Pathological stage, No. (%)			.121			.334			.147
Stage I	9,571 (45.6)	4,454 (46.9)		7,539 (48.3)	3,810 (48.2)		2,024 (37.9)	642 (40.3)	
Stage II	8,860 (42.2)	3,935 (41.4)		6,180 (39.5)	3,181 (40.2)		2,673 (50.1)	754 (47.3)	
Stage III	2,554 (12.2)	1,117 (11.7)		1,910 (12.2)	919 (11.6)		643 (12.0)	198 (12.4)	
pT stage, No. (%)^c^			.001			.041			.050
T1	12,522 (59.8)	5,801 (61.1)		9,967 (63.9)	5,001 (63.3)		2,546 (47.8)	798 (50.2)	
T2	7,594 (36.2)	3,365 (35.4)		5,078 (32.5)	2,631 (33.3)		2,510 (47.1)	734 (46.2)	
T3	688 (3.3)	291 (3.1)		467 (3.0)	240 (3.0)		220 (4.2)	51 (3.2)	
T4	146 (0.7)	35 (0.4)		97 (0.6)	28 (0.4)		49 (0.9)	7 (0.4)	
pN stage, No. (%)			.104			.213			.623
N0	13,702 (65.3)	6,118 (64.4)		10,047 (64.3)	5,048 (63.8)		3,643 (68.2)	1,068 (67.0)	
N1	4,978 (23.7)	2,376 (25.0)		3,856 (24.7)	2,034 (25.7)		1,119 (21.0)	342 (21.5)	
N2	1,430 (6.8)	636 (6.7)		1,094 (7.0)	536 (6.8)		335 (6.3)	100 (6.3)	
N3	875 (4.2)	376 (3.9)		632 (4.0)	292 (3.7)		243 (4.5)	84 (5.2)	
LNR, mean ± SD	0.085 ± 0.18	0.092 ± 0.20	.005	0.088 ± 0.18	0.092 ± 0.19	.104	0.077 ± 0.17	0.090 ± 0.20	.018
Histological type, No. (%)^d^			.007			.333			.059
Invasive ductal	19,683 (96.4)	8,942 (95.8)		14,552 (95.5)	7,406 (95.3)		5,122 (99.1)	1,534 (98.6)	
Invasive lobular	725 (3.6)	391 (4.2)		680 (4.5)	369 (4.7)		45 (0.9)	22 (1.4)	
Histological grade, No. (%)			.001			.001			.205
G1	4,232 (20.2)	1,831 (19.3)		4,029 (25.8)	1,770 (22.4)		200 (3.7)	61 (3.8)	
G2	8,430 (40.2)	4,437 (46.7)		7,341 (47.0)	4,081 (51.6)		1,087 (20.4)	355 (22.3)	
G3	6,534 (31.1)	2,500 (26.3)		2,940 (18.8)	1,473 (18.6)		3,591 (67.2)	1,027 (64.4)	
Unknown	1,789 (8.5)	738 (7.7)		1,319 (8.4)	586 (7.4)		462 (8.7)	151 (9.5)	
Nuclear grade, No. (%)			.001			.001			.783
G1	2,483 (11.8)	849 (8.9)		2,342 (15.0)	806 (10.3)		141 (2.6)	43 (2.7)	
G2	9,217 (44.0)	4,650 (48.9)		8,205 (52.5)	4,322 (54.6)		1,007 (18.9)	327 (20.5)	
G3	6,380 (30.4)	2,523 (26.6)		2,968 (19.0)	1,568 (19.8)		3,410 (63.9)	954 (59.9)	
Unknown	2,905 (13.8)	1,484 (15.6)		2,114 (13.5)	1,214 (15.3)		782 (14.6)	270 (16.9)	
Presence of LI, No. (%)^b^	5,231 (24.9)	2,712 (28.5)	.001	3,913 (25.0)	2,284 (14.6)	.001	1,316 (24.6)	428 (26.9)	.001
Presence of VI, No. (%)^b^	2,737 (13.0)	1,171 (12.3)	.148	2,048 (13.1)	960 (12.1)	.338	689 (12.9)	211 (13.2)	.202
Ki-67, No. (%)^b^									
< 14%	4,962 (48.2)	3,035 (58.7)	.001	4,415 (57.6)	2,793 (65.2)	.001	539 (20.7)	242 (27.4)	.001
≥ 14%	5,328 (51.8)	2,134 (41.3)		3,256 (42.4)	1,491 (34.8)		2,069 (79.3)	642 (72.6)	
< 20%	5,752 (55.9)	3,390 (65.6)	.001	5,115 (66.7)	3,095 (72.2)	.001	629 (24.1)	295 (33.4)	.001
≥ 20%	4,538 (44.1)	1,779 (34.4)		2,556 (33.3)	1,189 (27.8)		1,979 (75.9)	589 (66.6)	
Breast surgery, No. (%)^b^			.001			.004			.044
BCS	12,972 (62.5)	5,656 (60.3)		9,603 (62.1)	4,698 (60.2)		3,363 (63.6)	958 (60.8)	
TM	7,798 (37.5)	3,731 (39.7)		5,863 (37.9)	3,111 (39.8)		1,927 (36.4)	618 (39.2)	
Axillary surgery, No. (%)			.001			.001			.545
SLNB	6,111 (29.1)	3,236 (34.0)		4,616 (29.6)	2,765 (35.0)		1,494 (28.0)	470 (29.5)	
SLNB & ALND	4,177 (19.9)	2,366 (25.0)		3,196 (20.4)	2,023 (25.6)		980 (18.3)	343 (21.5)	
ALND	9,353 (44.6)	3,465 (36.4)		6,790 (43.4)	2,751 (34.8)		2,559 (48.0)	714 (44.8)	
No surgery	1,344 (6.4)	439 (4.6)		1,027 (6.6)	371 (4.7)		307 (5.7)	67 (4.2)	
Adjuvant RT, No. (%)^b^			.922			.747			.658
Yes	13,220 (69.1)	5,882 (69.2)		9,773 (68.5)	4,856 (68.7)		3,444 (71.0)	1,026 (71.6)	
No	5,907 (30.9)	2,621 (30.8)		4,497 (31.5)	2,212 (31.3)		1,407 (29.0)	407 (28.4)	
Adjuvant CT, No. (%)^b^			.002			.347			.141
Yes	13,979 (71.3)	6,099 (69.4)		9,466 (64.9)	4,774 (65.5)		4,509 (89.9)	1,325 (88.6)	
No	5,633 (28.7)	2,685 (30.6)		5,124 (35.1)	2,512 (34.5)		507 (10.1)	171 (11.4)	

*HR* hormone receptor; *HER2* human epidermal growth factor receptor 2; *HER2_0* HER2-IHC 0 breast cancer; *HER2_L* HER2-low breast cancer; *BMI* body mass index; *FHx* family history; *pT stage* pathological T stage; *pN stage* pathological N stage; *LNR* lymph node ratio; *SD* standard deviation; *LI* lymphatic invasion; *VI* vascular invasion; *BCS* breast conserving surgery; *TM* total mastectomy; *SLNB* sentinel lymph node biopsy; *ALND* axillary lymph node dissection; *RT* radiotherapy; *CT* chemotherapy

^a^Hormone receptor status was available for a total of 30,473 patients: 23,539 in the hormone receptor-positive group, and 6934 in the hormone receptor-negative group

^b^These variables have missing data

^c^The cases of T0 are not described here

^d^The cases of other histology are not described here

No significant differences were observed between adjuvant treatments administered according to HER2 status in HR-positive breast cancer and TNBC (Additional file 1: Table S1). Patients with TNBC received relatively more adjuvant chemotherapy than patients with HR-positive breast cancer. Anthracycline-containing regimens and selective estrogen receptor modulators were the most commonly used as adjuvant chemotherapy and endocrine therapy, respectively.

### Survival outcomes

**Fig. 2:**
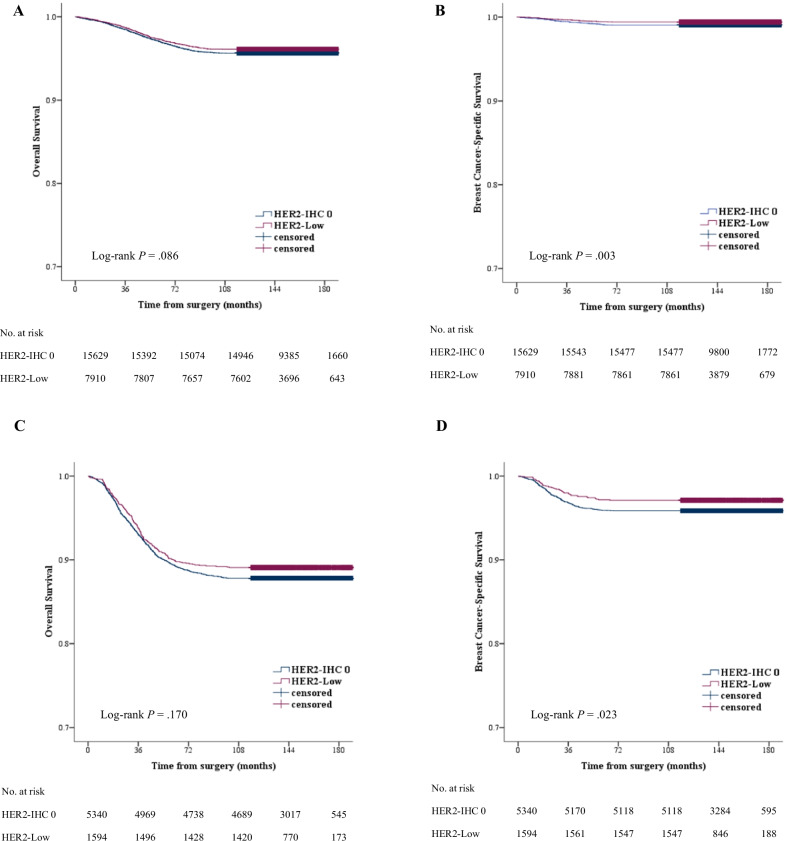
OS and BCSS (**A**, **B**) in HR-positive breast cancer and (**C**, **D**) in TNBC. No significant difference was noted in overall survival between patients with HER2-low breast cancer and those with HER2-IHC 0 breast cancer, but breast cancer-specific survival was significantly better in patients with HER2-low breast cancer than in those with HER2-IHC 0 breast cancer

**Fig. 3:**
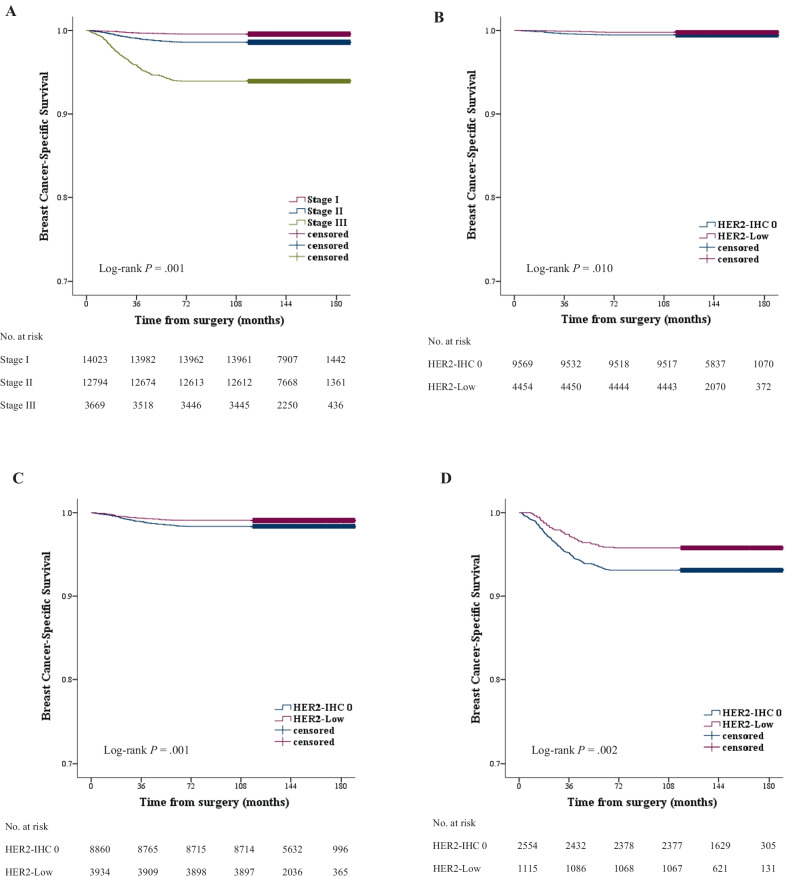
Kaplan–Meier survival curves for BCSS stratified by pathological stage. **A** A significant association was observed between pathological stage and breast cancer-specific survival. Breast cancer-specific survival was significantly better in patients with HER2-low breast cancer than in those with HER2-IHC 0 breast cancer in stages I (**B**), II (**C**), and III (**D**)

The median follow-up time was 148.0 months (range, 0.5–189.6 months). During the follow-up period, 1,817 cases of death from any cause and 465 cases of death caused by breast cancer were noted in all patients. Overall, patients with TNBC had worse OS (HRs, 2.98; 95% CI, 2.72–3.27; *P* < 0.001) and BCSS (HRs, 4.66; 95% CI, 3.88–5.60; *P* < 0.001) than patients with HR-positive breast cancer. The 5-year OS rates for the patients with HR-positive breast cancer and TNBC were 97.1% and 89.6%, respectively. The 5-year BCSS rates for the patients with HR-positive breast cancer and TNBC were 99.2% and 96.2%, respectively. Regarding the Kaplan–Meier survival curves, no significant differences were observed in OS between the HER2-low and HER2-IHC 0 groups for both HR-positive breast cancer and TNBC (*P* = 0.086 and 0.170, respectively) (Fig. 2a, c). However, significantly better BCSS was observed in the HER2-low group than the HER2-IHC 0 group for both HR-positive breast cancer and TNBC (*P* = 0.003 and 0.023, respectively) (Fig. 2b, d). For HR-positive breast cancer, the 5-year BCSS rates for HER2-low and HER2-IHC 0 groups were 99.4% and 99.1%, respectively. For TNBC, the 5-year BCSS rates for HER2-low and HER2-IHC 0 groups were 97.2% and 95.9%, respectively. We conducted a survival analysis according to the pathological stage. A significant association was found between the pathological stage and BCSS, i.e., diagnosis at an earlier stage was associated with better BCSS (*P* = 0.001). BCSS for HER2-low breast cancer at stages I to III was better than for HER2-IHC 0 breast cancer at each corresponding stage. (*P* = 0.010, 0.001, and 0.002, respectively) (Fig. 3).

The univariate and multivariate analyses of clinicopathological factors for BCSS for HR-positive breast cancer and TNBC are summarized in Table 2. For HR-positive breast cancer, advanced pathological stage, tumor size ≥ 5 cm, high LNR, histological grade 3, presence of lymphatic invasion or vascular invasion, high Ki-67 labeling index, and HER2-IHC 0 were associated with worse BCSS in the univariate analysis. In the multivariate analysis, advanced pathological stage, and histological grade 3 were associated with worse BCSS. For TNBC, advanced pathological stage, tumor size ≥ 5 cm, high LNR, presence of lymphatic invasion or vascular invasion, and HER2-IHC 0 were associated with worse BCSS in the univariate analysis. In the multivariate analysis, advanced pathological stage, tumor size ≥ 5 cm, high LNR, presence of lymphatic invasion, and HER2-IHC 0 were associated with worse BCSS.

**Table 2:** Univariate and multivariate analyses for breast cancer-specific survival

Variables	Univariate analysis		Multivariate analysis	
	HRs (95% CI)	*P* value	HRs (95% CI)	*P* value
**A. Hormone receptor-positive**				
Pathological stage		.001		.001
Stage I	1.00		1.00	
Stage II	2.81 (1.82–4.34)		1.81 (0.87–3.76)	
Stage III	14.34 (9.50–21.67)		5.99 (2.34–15.34)	
Tumor size: > 5 vs. ≤ 5 cm	6.82 (4.81–9.68)	.001	1.59 (0.83–3.03)	.159
LNR: Above vs. Below mean	4.10 (3.07–5.47)	.001	1.24 (0.64–2.38)	.527
Histological grade: G3 vs. G1/2	4.20 (3.14–5.60)	.001	3.41 (2.04–5.69)	.001
Lymphatic invasion: Yes vs. No	3.28 (2.44–4.41)	.001	1.65 (0.94–2.89)	.079
Vascular invasion: Yes vs. No	2.70 (1.94–3.75)	.001	0.79 (0.42–1.49)	.471
Ki-67: < 14 vs. ≥ 14%	0.54 (0.34–0.84)	.006	0.95 (0.57–1.56)	.825
HER2: Low vs. IHC 0	0.61 (0.44–0.85)	.003	0.72 (0.44–1.17)	.186
**B. Hormone receptor-negative**				
Pathological stage		.001		.001
Stage I	1.00		1.00	
Stage II	2.80 (1.89–4.14)		2.03 (1.35–3.05)	
Stage III	12.95 (8.77–19.13)		3.95 (2.34–6.63)	
Tumor size: > 5 vs. ≤ 5 cm	5.38 (3.97–7.29)	.001	1.94 (1.38–2.74)	.001
LNR: Above vs. Below mean	4.89 (3.83–6.26)	.001	1.92 (1.37–2.67)	.001
Histological grade: G3 vs. G1/2	1.29 (0.95–1.73)	.102		
Lymphatic invasion: Yes vs. No	4.27 (3.29–5.53)	.001	1.74 (1.23–2.46)	.006
Vascular invasion: Yes vs. No	3.88 (2.96–5.09)	.001	1.38 (0.99–0.19)	.132
Ki-67: < 14 vs. ≥ 14%	0.90 (0.54–1.49)	.691		
HER2: Low vs. IHC 0	0.69 (0.50–0.95)	.023	0.68 (0.49–0.93)	.019

To reduce the effect of other confounding factors that affect survival, we performed an additional analysis using IPTW. Potential confounding variables were balanced between patients with HER2-low breast cancer and those with HER2-IHC 0 breast cancer after IPTW (Additional file 2: Table S2). No significant difference was observed in impact of low HER2 expression on BCSS before and after IPTW (Table 3). In the IPTW-adjusted cohort, low HER2 expression showed a trend towards better BCSS for TNBC (log-rank test *P* = 0.079, Additional file 3: Figure S1).

**Table 3:** The impact of HER2-low expression on breast cancer-specific survival

	Breast cancer-specific survival			
	Before IPTW		After IPTW	
	HRs (95% CI)	*P* value	HRs (95% CI)	*P* value
Hormone receptor-positive	0.72 (0.44–1.17)	.188	0.72 (0.44–1.17)	.187
Hormone receptor-negative	0.64 (0.38–1.09)	.098	0.61 (0.35–1.05)	.075

Additionally, we divided HER2-low breast cancer into HER2 IHC 1 + and 2 + /ISH negative group. When comparing BCSS according to the HER2 IHC score (0, 1 + , and 2 + /ISH negative), there was a trend for improved survival in proportion to the HER2 expression levels, in both HR-positive breast cancer and TNBC (Additional file 4: Figure S2).

## Discussion

In the current clinical practice, HER2-low breast cancer is classified either as HR-positive breast cancer or TNBC, and the presence of low HER2 expression is not considered a factor in treatment decision-making. In addition, several previous studies reported on the prognostic role of low HER2 expression, but their conflicting findings did not provide robust evidence for low HER2 expression being an independent prognostic factor for breast cancer [12–15]. However, the status of HER2-low breast cancer has undergone a paradigm shift as a result of recent basic and clinical research findings from two perspectives. First, with the emergence of novel anti-HER2 antibody–drug conjugates, potential practice-changing clinical trials for HER2-low breast cancer are ongoing. Second, HER2-low breast cancer may be a clinically and biologically unique disease entity and may affect the prognosis of patients [11, 16]. Over the past decades, many advances in molecular biology and genomics have occurred leading to the identification of intrinsic molecular subtypes of breast cancer, and genetic information obtained from big data, such as The Cancer Genome Atlas (TCGA) data, has allowed a growing understanding of tumor biology and heterogeneity [17]. Although evidence to date is insufficient to reach solid conclusions, interesting data supporting HER2-low breast cancer as a new disease entity with distinct characteristics have been reported [7, 18–20]. Our study based on a large nationwide database also supports this point of view and provides insights into the biology of HER2-low breast cancer.

Our findings suggested that the clinical impact of low HER2 expression may differ depending on the HR status. HER2-low breast cancer was more common in patients with HR-positive breast cancer than in those with TNBC, and the clinicopathological characteristics of HER2-low breast cancer showed a slightly different pattern depending on the HR status. Among patients with HR-positive breast cancer, HER2-low breast cancer was more frequent in premenopausal patients and associated with fewer T4 tumors, higher histological grade, and a negative lymphatic invasion compared with HER2-IHC 0 breast cancer. Among patients with TNBC, HER2-low breast cancer was more frequent in older patients and associated with a high LNR and positive lymphatic invasion compared with HER2-IHC 0 breast cancer. Across subtypes, HER2-low breast cancer was more frequent in overweight patients with BMI ≥ 25 kg/m^2^ and those with Ki-67 labeling index < 14% or 20% than HER2-IHC 0 breast cancer. Schettini et al. recently reported the clinical features of HER2-low breast cancer using the clinicopathological and PAM50 gene expression data from 3,689 patients with HER2-negative breast cancer [19]. They reported that HER2-low breast cancer was more frequent in older and male patients and associated with more axillary LNs involvement compared with HER2-IHC 0 breast cancer. In analyses of PAM50 intrinsic subtypes according to HER2 status (n = 1,576), luminal A subtype (58.9% vs. 51.8%) was more frequent and luminal B (33.4% vs. 34.9%) and basal-like subtypes (1.9% vs. 8.0%) were less frequent in HER2-low breast cancer compared with HER2-IHC 0 breast cancer among patients with HR-positive breast cancer. However, no significant difference was observed in the subtype distribution between HER2-low and HER2-IHC 0 breast cancer among patients with TNBC. Consistent with this intrinsic subtype analysis, individual gene expression data showed that proliferation-related genes were significantly downregulated and luminal-related genes were upregulated in HER2-low breast cancer compared with HER2-IHC 0 breast cancer. These findings may also explain our result that Ki-67 labeling index < 14% was more frequent in HER2-low breast cancer than in HER2-IHC 0 breast cancer. Analyses of the clinical characteristics of HER-low breast cancer from four prospective neoadjuvant clinical trials showed that HER2-low breast cancer was significantly associated with a lower number of G3 tumors, lower Ki-67 labeling index, and reduced number of TP53 mutations compared with HER2-IHC 0 breast cancer [18]. Agostinetto et al. reported slightly different findings regarding the distribution of PAM50 intrinsic subtypes between HER2-low and HER2-IHC 0 breast cancer using TCGA dataset [20, 21]. Significant differences in the distribution of intrinsic subtypes were reported between the two groups in patients with TNBC, but not in those with HR-positive breast cancer. Among patients with TNBC, basal-like breast cancer was the most common subtype in both HER2-low and HER2-IHC 0 breast cancer, but HER2-low breast cancer was characterized by a higher proportion of HER2-enriched subtypes compared with HER2-IHC 0 breast cancer (13.7% vs. 1.6%). Taken together, to date, not all studies on the characteristics of HER2-low breast cancer have produced consistent results, and in our analysis of the clinicopathological factors associated with HER2-low status, mixed results with good and poor prognostic factors were obtained. Nevertheless, these data indicate that HR status has a crucial role among patients with HER2-low breast cancer, and this indication is in line with the different response rates of novel anti-HER2 antibody–drug conjugates for HER2-low breast cancer according to the HR status [8, 10]. In addition, these findings support that a difference can exist in the intrinsic molecular subtypes between HER2-low and HER2-IHC 0 breast cancer, and this can contribute to different clinical behaviors and prognoses.

Regarding the impact of low HER2 expression on survival outcomes, our findings showed no significant difference in OS between the HER2-low and HER2-IHC 0 groups, but the former group had significantly better BCSS than the latter group in case of both HR-positive breast cancer and TNBC. However, in multivariate analysis, the impact of low HER2 expression on BCSS was significant only in case of TNBC. After applying the IPTW, there was a similar trend for the impact of low HER2 expression on BCSS. These findings of association between low HER2 expression and favorable outcome were consistent with recent results. Denkert et al. reported the difference in disease-free survival (DFS) and OS between HER2-low and HER2-IHC 0 breast cancer using data from four prospective neoadjuvant clinical trials. Patients with HER2-low breast cancer had a significantly longer DFS and OS than did those with HER2-IHC 0 breast cancer in case of TNBC [18]. Moreover, a trend, though not statistically significant, toward prolonged DFS and OS was observed for HER2-low breast cancer in patients with HR-positive breast cancer. Mutai et al. analyzed data from 608 patients with HR-positive early breast cancer, and found that HER2-low breast cancer was associated with significant improvement in OS and DFS compared with HER-IHC 0 breast cancer in patients with high genomic risk [22]. Schettini et al. analyzed overall survival data from 1,304 patients with metastatic breast cancer, and reported that there was no significant difference in OS between HER2-low and HER2-IHC 0 breast cancer in case of both HR-positive breast cancer and TNBC [19]. To date, no clear mechanism exists to explain why patients with HER2-low tumors have better survival outcomes than those with HER2-IHC 0 tumors in early breast cancer. This could be attributed to a difference in the intrinsic subtypes between HER2-low and HER2-IHC 0 breast cancer; factors related to reduced aggressiveness, such as low Ki-67 labeling index; the existence of more complex unknown biology related to HER2-low breast cancer. Although our results showed potential association between low HER2 expression and favorable outcome, it was a small difference numerically that further research is needed to see if it has any clinical significance. Further studies including molecular profiling, broader genomic analysis, and functional studies of HER2-low breast cancer are warranted to address these unclear aspects regarding this breast cancer subtype. In addition, results in early breast cancer may not be consistent with those in metastatic breast cancer. Therefore, further studies on differences in the role of low HER2 expression between early and metastatic breast cancer in terms of response to therapy and prognosis are needed.

This study has some limitations. First, this was a retrospective study related to potential biases. Since patients without HER2 ISH records were excluded, the proportion of patients with HER2-low breast cancer was relatively lower in this study. Second, evaluation of ER, PR, and HER2 status were assessed from local pathology reports and no central confirmation of pathological assessment performed. Although all institutions are following the guidelines of the American Society of Clinical Oncology/College of American Pathologists, the reproducibility, especially in the IHC score of 0 or 1 + for HER2, cannot be guaranteed [19]. Third, as information related to recurrence was not included in the KBCR database, no analysis was conducted on this. Finally, most of the patients included in this study were Korean, and only 252 patients had foreign nationality. Since there may be racial and ethnic differences in the biology of cancer, it is necessary to be careful in generalizing these results.

## Conclusion

The concept of HER2 status is evolving with the emergence of new therapeutic strategies in HER2-low breast cancer. Our findings using large nationwide data strengthen the rationale that it is necessary to consider the HR status in the HER2-low category and the possibility of biological differences between HER2-low and HER2-IHC 0 breast cancer. However, it seems that we need to wait for more research data to conclude that HER2-low breast cancer is a new biologic subtype with different prognosis. Unmet needs still exist regarding improvement of the prognosis for HER2-low breast cancer and more efforts are required for a deeper understanding of HER2-low breast cancer in future.

## Supplementary Information

**Additional file 1: Table S1**. Adjuvant treatments according to HER2 status within hormone receptor-positive and triple-negative breast cancer.

**Additional file 2: Table S2**. Patients characteristics according to HER2 status before and after using IPTW.

**Additional file 3: Figure S1**. Kaplan–Meier survival curves for OS and BCSS in hormone receptor-positive breast cancer (A, B) and in triple-negative breast cancer (C, D) after IPTW.

**Additional file 4: Figure S2**. Forest plot with hazard ratio showing BCSS according to HER2 IHC score in hormone receptor-positive breast cancer (A) and in triple-negative breast cancer (B).

## Data Availability

The dataset used and analyzed during the current study is available from the corresponding author on reasonable request.

## Abbreviations

HER2: Human epidermal growth factor receptor 2
IHC: Immunohistochemistry
ISH: In situ hybridization
HR: Hormone receptor
KBCR: Korean Breast Cancer Registry
BMI: Body mass index
LNs: Lymph nodes
ER: Estrogen receptor
PR: Progesterone receptor
LNR: Lymph node ratio
OS: Overall survival
BCSS: Breast cancer-specific survival
HRs: Adjusted hazard ratios
CI: Confidence intervals
IPTW: Inverse probability of treatment weighting
TNBC: Triple-negative breast cancer
TCGA: The Cancer Genome Atlas
