# Supplementary material for: Clinical significance of HER2-low expression in early breast cancer: a nationwide study from the Korean Breast Cancer Society
Source: Breast Cancer Res. 2022 Mar 21;24:22. doi: 10.1186/s13058-022-01519-x (PMC8935777; doi:10.1186/s13058-022-01519-x)
Supplement: Supplementary file 1 — Additional file 1: Table S1. Adjuvant treatments according to HER2 status within hormone receptor-positive and triple-negative breast cancer. [file 13058_2022_1519_MOESM1_ESM.docx]

Table S1. Adjuvant treatments according to HER2 status within hormone receptor-positive and triple-negative breast cancer

|  | HR-positive breast cancer  (N = 21,877) | *P* | Triple-negative breast cancer  (N = 6,512) | *P* |
| --- | --- | --- | --- | --- |

|  | HER2 IHC 0 | HER2-Low |  | HER2 IHC 0 | HER2-Low |  |
| --- | --- | --- | --- | --- | --- | --- |

| Adjuvant Chemotherapy |
| --- |

| No Treatment | 5,124 (35.1) | 2,512 (34.5) | 0.068 | 507 (10.1) | 171 (11.4) | 0.642 |
| --- | --- | --- | --- | --- | --- | --- |
| CMF | 1,773 (12.2) | 688 (9.4) |  | 604 (12.0) | 166 (11.1) |  |
| AC | 3,120 (21.4) | 1,868 (25.6) |  | 2,175 (43.4) | 661 (44.2) |  |
| AC followed by taxane | 3,701 (25.3) | 1,895 (26.0) |  | 1,220 (24.3) | 353 (23.6) |  |
| Others | 873 (6.0) | 323 (4.5) |  | 510 (10.2) | 145 (9.7) |  |

| Adjuvant Endocrine therapy |
| --- |

| SERMs | 8,214 (56.3) | 3,782 (52.0) | 0.051 |  |  |  |
| --- | --- | --- | --- | --- | --- | --- |
| AIs | 3,771 (25.8) | 1,709 (23.5) |  |  |  |  |
| SERMs 🡪 AIs | 141 (1.0) | 49 (0.7) |  |  |  |  |
| Unknown | 2,465 (16.9) | 1,746 (23.8) |  |  |  |  |

CMF, cyclophosphamide, methotrexate, 5-fluorouracl; AC, anthracycline, cyclophosphamide; SERMs, selective estrogen receptor modulators; AIs, aromatase inhibitors.
