# Supplementary material for: Clinical significance of HER2-low expression in early breast cancer: a nationwide study from the Korean Breast Cancer Society
Source: Breast Cancer Res. 2022 Mar 21;24:22. doi: 10.1186/s13058-022-01519-x (PMC8935777; doi:10.1186/s13058-022-01519-x)
Supplement: Supplementary file 4 — Additional file 4: Figure S2. Forest plot with hazard ratio showing BCSS according to HER2 IHC score in hormone receptor-positive breast cancer (A) and in triple-negative breast cancer (B). [file 13058_2022_1519_MOESM4_ESM.pptx]

## Slide 1
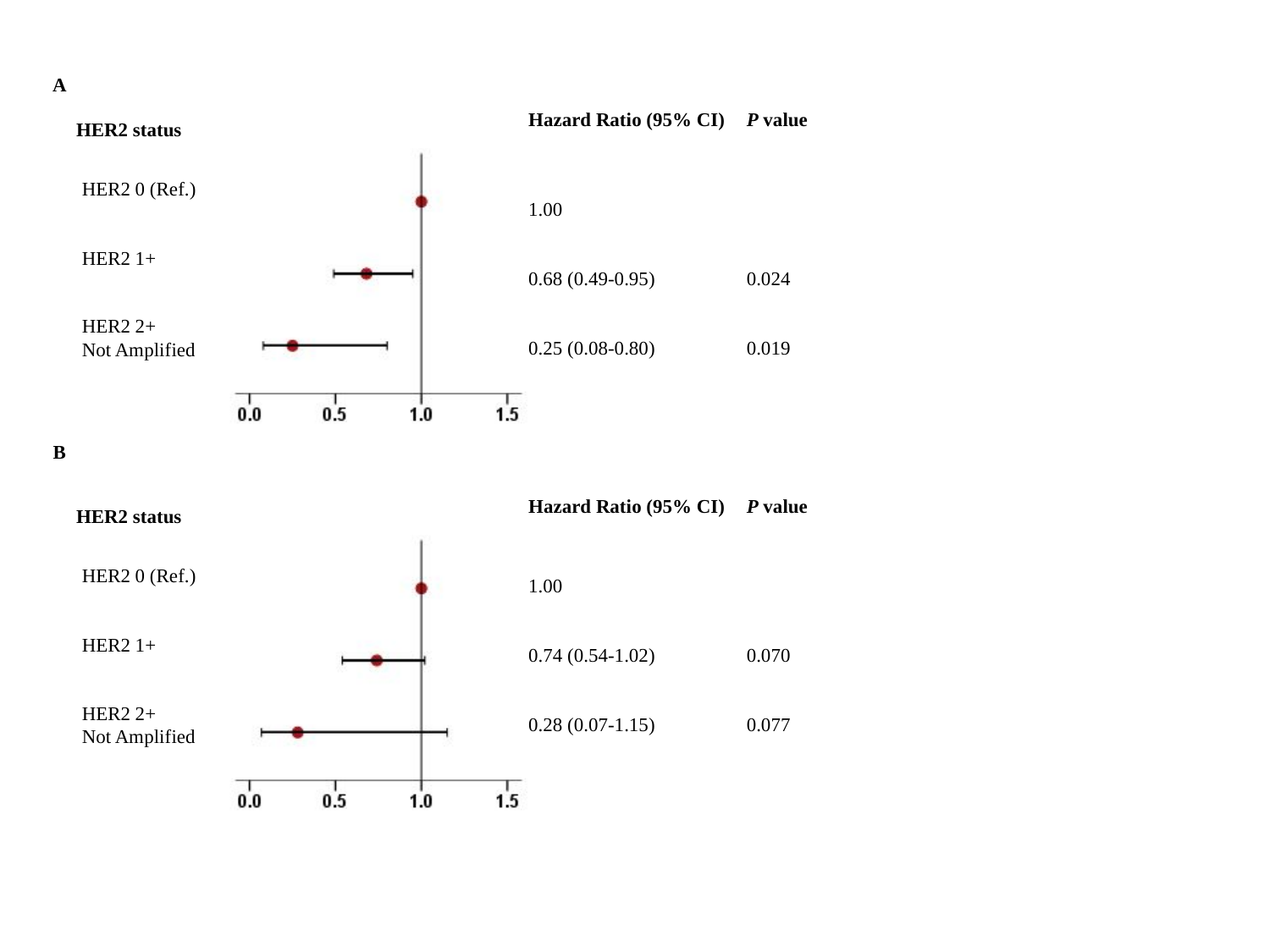

A
Hazard Ratio (95% CI)
P value
HER2 status
HER2 0 (Ref.)
1.00
HER2 1+
0.68 (0.49-0.95)
0.024
HER2 2+
Not Amplified
0.25 (0.08-0.80)
0.019
B
Hazard Ratio (95% CI)
P value
HER2 status
HER2 0 (Ref.)
1.00
HER2 1+
0.74 (0.54-1.02)
0.070
HER2 2+
Not Amplified
0.28 (0.07-1.15)
0.077
